# Predictors of intention to provide abortions after OB/GYN residency training

**DOI:** 10.1371/journal.pone.0286703

**Published:** 2023-06-29

**Authors:** Katherine J. Kramer, Sarah Ottum, Conrad R. Chao, Aliye Runyan, Benjamin Rappolee, Sandra Sadek, Noor E. Jannat, Maurice-Andre Recanati

**Affiliations:** 1 Department of Obstetrics and Gynecology, St. Vincent’s Medical Center, Manhattan, New York, United States of America; 2 Department of Obstetrics and Gynecology, University of Cincinnati, Cincinnati, Ohio, United States of America; 3 Department of Obstetrics and Gynecology, University of New Mexico, Albuquerque, New Mexico, United States of America; 4 Department of Obstetrics and Gynecology, Westchester Medical Center, Valhalla, New York, United States of America; 5 Post Baccalaureate Student, Oakland University, Rochester, Michigan, United States of America; 6 Department of Obstetrics, Gynecology and Reproductive Sciences, University of Texas Health Science Center, Houston, Texas, United States of America; 7 Department of Computer Science, Wayne State University, Detroit, Michigan, United States of America; 8 Department of Obstetrics and Gynecology, Wayne State University, Detroit, Michigan, United States of America; United Arab Emirates University, UNITED ARAB EMIRATES

## Abstract

**Introduction:**

Abortion is a common gynecological procedure and plays a central role in women’s health and autonomy. To maintain accessibility to abortion, it is important that sufficient obstetrics and gynecology (Ob/Gyn) residents intend to provide abortion care after residency. This study identifies factors that influence a resident’s intention to provide abortions (IPA) post-training.

**Materials and methods:**

A multiple-choice survey, addressing demographics, religious background, residency program metrics, training experience and intent to provide abortions (IPA), was answered by 409 Ob/Gyn residents. Chi-square test was performed on descriptive statistics and continuous variables were tested with ANOVA with p<0.05 considered significant.

**Results:**

Residents with IPA were predominantly female (p = 0.001), training in the Northeast and West (p<0.001), identifying either as non-religious, agnostic/atheist or Jewish (p<0.01), not actively practicing their religion (p<0.001) and leaning democrats (p<0.002). Those with IPA were more likely to train at hospitals without religious affiliation (p<0.008), to train at a Ryan Program (p<0.001), to place strong emphasis on choosing a program with family planning training (p<0.001), to join programs where a significant portion of the faculty performs abortions (p<0.001) and to have completed a higher number of first trimester medical and surgical abortion procedures during the last six months of training (p<0.001).

**Conclusion:**

These results suggest that factors influencing a physician’s intention to provide abortions are multifactorial, involving personal and program factors. A model predicting IPA is derived. To maximize IPA, residency programs can increase abortion volume, facilitate additional training and build a supportive faculty.

## Introduction

Abortion is one of the most common gynecological procedures and plays a central role in women’s health and body autonomy [1, 2]. In the United States, about half of all pregnancies are unintended [3] and about half of those result in abortion [4]. For abortion to remain available and accessible [5] it must remain affordable [6], legal [7], and residency programs must teach competency in family planning techniques. Residents must also have the intention of providing abortions (IPA) post training and future providers may have to negotiate significant barriers to performing abortions as attendings, especially given the continually evolving political climate in the United States.

This study seeks to identify the factors that influence an OB/GYN resident’s IPA post-training; however, analyzing barriers which may subsequently limit a physician’s ability to actually provide such care are beyond the scope of this paper. The effects of the legal environment in the state that the resident is training, the presence of a Ryan program, the training environment (in terms of case numbers), the hospital setting (religious vs non-religious) and the personal demographics (religiosity, political leanings, age) of the individual resident are examined in order to determine their influence on the intention to provide abortion.

## Materials and methods

### Electronic survey

This study was approved by the Wayne State IRB under expedited review (https://waynestate.az1.qualtrics.com/jfe/form/SV_7VssqjccfquwnVb). A pre-tested anonymous CHERRIES-compliant Qualtrics (Qualtrics International, Provo, UT, USA) survey, S1 File, was sent to each Obstetrics and Gynecology residency training program director in the United States with a request to forward the survey to their residents. All participants were adult physician-residents holding a doctor of medicine (M.D.) or doctor of osteopathic medicine (D.O.). The multiple-choice survey page stated that participation was voluntary, that taking part in the survey constituted written consent to participate, gave permission not to answer specific questions and to withdraw at any time. All data was deidentified and anonymized at the time of data collection. It consisted of the following sections: demographics, religious background, political views, residency program metrics and intent to provide. IPA, the independent variable, was analyzed as a binary value (0: no intent, 1: intent to provide abortions in any trimester). States were grouped into regions as defined by the U.S. Census Bureau [8] and size of area of origin was grouped into three groups (1: areas with > ½ a million residents, 2: cities of 100–500 thousands and 3:rural and small urban areas). Political affiliation was grouped as Republican and Democrat based on choice of elected candidate. Religion was grouped into Catholics, all Christian denominations, Atheists/Agnostics, Jewish and Other which encompassed a small number of Hindu (n = 13), Buddhist (n = 3), Muslim (n = 4) and those identifying as “other” (n = 19) on the survey. It was analyzed both as categorical and as continuous data based on a scale of degree of conservatism as described by Pew Research [9]. Questions involving case numbers (contraceptive visits, tubal sterilizations, IUD and Nexplanon insertions, medical and surgical abortions) were analyzed as continuous data. Similarly, percentage of faculty performing abortions was grouped into a trinary scale (0: no faculty does abortions, 1: <50%, 2: 50% or more).

### Statistical analysis

Qualtrics data was downloaded into an encrypted Excel spreadsheet, deidentified through the removal of IP addresses and analyzed using Statistical Package for the Social Sciences (SPSS) (IBM Analytics). Descriptive statistics were used to characterize the sample. Pearson Chi-square test was performed in order to test the association between one categorical variable and another. A value of p<0.05 was considered significant. Binary dependent variables were treated as continuous so two-tailed t-tests could be performed. Continuous dependent variables were tested with ANOVA and post-hoc testing was used (with Bonferroni correction) to identify categories that showed significant differences. The tables note the significantly different pairs, with letters indicating the category row that has the significantly lower value and the p-value in parenthesis. A binary stepwise logistic regression model was established to predict IPA. The routine allows variables to be added to the regression if the F-test shows an improvement at p<0.05, and removes variables if removal shows p<0.10.

## Results

### Description of the cohort

#### Description of the residents

In the United States, there are 296 OB/GYN programs [10] training 5,563 residents [11]. The survey was answered by 422 residents of which 13 surveys were excluded as they were completed from programs outside the U.S., leaving n = 409. The cohort of residents, Table 1, was predominantly female, under 30 years old, heterosexual, married, and Democratic. The cohort tended to be atheist/agnostic or did not adhere to a religion while those that did were mostly Catholic or non-denominational. While a majority of residents came from practicing families with both parents regularly attending services, only 16% of residents continued to attend regularly while 50% did not practice at all. Resident’s region of origin was evenly distributed geographically and about 4% of respondents considered themselves to be originally from outside the country but undergoing residency in the United States. A majority of OB/GYN residents came from large cities while a quarter came from rural areas. All four years of residency training were represented equally.

**Table 1:** Cohort of residents.

		Count	Column N %
**Age**	25–30	230	56%
	31–35	165	40%
	36–45	12	3%
	46+	1	0%
**Gender**	Female	362	89%
	Male	45	11%
	Trans/Intersex/Nonbinary	1	0%
**Sexual Orientation**	Heterosexual	370	92%
	Bisexual	19	5%
	Homosexual	15	4%
**Marital Status**	Married	210	52%
	Single	90	22%
	Long term relationship	46	11%
	Engaged	29	7%
	Dating	30	7%
	Decline to answer	2	0%
**Region of Origin**	Midwest	112	28%
	West	101	25%
	South	92	23%
	Northeast	82	20%
	Outside US	16	4%
**Size of Origin**	Major metro area of 1mill+	121	30%
	Large city of 0.5-1mill	49	12%
	City of 100,000–0.5mill	82	20%
	Small urban area of 50–100,000	64	16%
	Rural (less than 50,000)	91	22%
**Region of Residency**	Midwest	125	31%
	Northeast	112	28%
	West	105	26%
	South	65	16%
**Year of Training**	PGY1	101	25%
	PGY2	100	25%
	PGY3	110	27%
	PGY4	97	24%
**Current Religion**	Christian (all denominations)	98	24%
	Atheist/Agnostic	90	22%
	No religion identified	77	19%
	Catholic	76	19%
	Other	39	10%
	Jewish	28	7%
**Family’s Degree of Religiosity**	Yes, both parents attend services regularly	186	46%
	Parents only attend during major holidays	110	27%
	Parents are not practicing religion but do believe	53	13%
	Parents non-religious/ Not at all	57	14%
**Current Religious Practice**	Not at all	202	50%
	Only on major holidays	140	34%
	Actively (attend daily/weekly)	65	16%
**Political Party Affiliation**	Democratic	358	88%
	Republican	34	8%
	No Answer	17	4%

#### Description of the training environment

When matching, the majority of residents considered it extremely or very important that the program offered family planning training. A majority of respondents were training at non-religiously affiliated institutions with a Ryan program and an “opt-out” of family planning training policy. For those who chose to opt-out, the majority of programs (91%) did not stigmatize those residents (Table 2). About 66% of programs offered formal didactic teaching on options counseling while 29% were taught in the clinic. About 10% of training programs had no faculty performing abortions.

**Table 2:** The residency training environment.

		Column N %	Count
**Hospital Religious Affiliation**	Not religiously affiliated/none	83%	339
	Christian/Adventist/ Baptist/Catholic	15%	61
	Jewish	1%	6
**Importance of Family Planning in Choosing Program**	Extremely important	37%	152
	Very important	25%	101
	Moderately important	19%	77
	Slightly important	9%	36
	Not at all important	10%	39
	Chose because did NOT	1%	3
**Residency a Ryan Program?**	Yes	68%	263
	No	24%	95
	Unsure	8%	30
**Opt-In or Opt-Out Program**	Opt-Out	89%	348
	Opt-In	11%	41
**Stigma in Opt-Out?**	No	91%	353
	Yes	9%	35
**Abortion Training Experience**	At Least Some In-House	84%	342
	Only Outside	16%	67
**Fraction of Faculty Provide Abortions**	None (0%)	11%	41
	Only a few (20%)	46%	180
	About half (50%)	26%	103
	Majority (80%)	17%	65
**Training Tubal Cases (past 6 months)**	None (0)	2%	9
	1–5 (3)	28%	108
	6–10 (8)	33%	128
	11–20 (16)	22%	87
	>21 (30)	15%	57
**Training IUD Cases (past 6 months)**	None (0)	1%	3
	1–5 (3)	24%	95
	6–10 (8)	33%	130
	11–20 (16)	25%	97
	>21 (30)	16%	64
**Training Nexplanon Cases (past 6 months)**	None (0)	4%	14
	1–5 (3)	36%	141
	6–10 (8)	26%	102
	11–20 (16)	19%	72
	>21 (30)	15%	60
**First Trimester Medical Cases (past 6 months)**	None (0)	36%	138
	1–5 (3)	33%	128
	6–10 (8)	14%	53
	11–20 (16)	10%	39
	>21 (30)	7%	29
**First Trimester Surgical Cases (past 6 months)**	None (0)	23%	90
	1–5 (3)	28%	108
	6–10 (8)	16%	62
	11–20 (16)	18%	70
	>21 (30)	15%	58
**Surgical Cases up to 18 weeks (past 6 months)**	None (0)	32%	126
	1–5 (3)	40%	154
	6–10 (8)	14%	55
	11–20 (16)	9%	35
	>21 (30)	5%	18
**Surgical Cases up to 23 weeks (past 6 months)**	None (0)	48%	187
	1–5 (3)	34%	130
	6–10 (8)	9%	36
	11–20 (16)	5%	20
	>21 (30)	4%	15
**Surgical Cases to term (past 6 months)**	None (0)	89%	341
	1–5 (3)	9%	33
	6–10 (8)	1%	4
	11–20 (16)	1%	2
	>21 (30)	1%	4
**How many contraceptive visits seen (past 6 months)?**	None (0)	1%	3
	1–5 (3)	8%	30
	6–10 (8)	18%	70
	11–20 (16)	20%	78
	>21 (30)	53%	208
**Options Counseling Training?**	Not at all	20	5%
	Only as needed in the clinic	113	29%
	Yes, in formal didactics	256	66%
**Sought Out Additional Family Planning Training Outside?**	No	349	90%
	Yes	40	10%
**OBGYN Career Choice Factor—diversity of clinical settings**	No	39	10%
	Yes	370	90%
**OBGYN Career Choice Factor—female patient population**	No	160	39%
	Yes	249	61%
**OBGYN Career Choice Factor—primary care specialty**	No	344	84%
	Yes	65	16%
**OBGYN Career Choice Factor—surgical specialty**	No	94	23%
	Yes	315	77%
**OBGYN Career Choice Factor—social aspects of care**	No	210	51%
	Yes	199	49%
**Second Trimester Method–Dilation and Evacuation**	No	89	22%
	Yes	320	78%
**Second Trimester Method—Induction**	No	178	44%
	Yes	231	56%
**Second Trimester Method—Referral**	No	336	82%
	Yes	73	18%
**Plan to be Abortion Provider**	No	126	33%
	Yes, only medical abortion	9	2%
	Yes, first trimester procedures	91	24%
	Yes, up to second trimester after additional training	54	14%
	Yes, second trimester procedures	107	28%

### Intention to provide abortion post residency

We compared those with stated intention to provide abortions to those without such intentions (Table 3). Intent was scored in a binary fashion and this question was answered by n = 387 respondents. Female residents were significantly more likely to intend to provide abortions (p = 0.001) when compared to their male colleagues. A resident’s region of origin (p = 0.008), region of residency training (p<0.001), political leaning (p<0.002) and religion (p<0.001) were associated with IPA. Atheists/agnostics and Jewish residents as well as those not practicing any religion were more likely to IPA when compared to their Catholic (p<0.002) and Christian (p<0.002) colleagues, even when accounting for gender. The religious environment into which residents matured, their current religious practice and their political leanings also had an impact on IPA (p<0.001). Those from families where both parents attend services regularly were less likely to IPA and, conversely, those who were not religious were more likely to provide when compared to their peers from other backgrounds (p<0.001).

**Table 3:** Intention to provide abortions.

		Count	Intend to Perform Abortions	p-value across rows
**Gender p = 0.001**	Female (A)	362	**70%**	B(0.001)
	Male (B)	45	43%	
**Marital Status NS**	Married	210	63%	
	Long term relationship	46	79%	
	Engaged	29	75%	
	Dating	30	61%	
	Single	90	71%	
	Decline to answer	2	100%	
**Sexual Orientation NS**	Heterosexual	370	67%	
	Bisexual	19	74%	
	Homosexual	15	54%	
**Region of Origin p = 0.008**	Outside US (A)	16	69%	
	Northeast (B)	82	**83%**	C(0.022) D(0.007)
	Midwest (C)	112	61%	
	South (D)	92	58%	
	West (E)	101	70%	
**Size of Origin NS**	Major metro area of 1mill+	121	66%	
	Large city of 0.5-1mill	49	83%	
	City of 100,000–0.5mill	82	70%	
	Small urban area of 50–100,000	64	65%	
	Rural (less than 50,000)	91	60%	
**Region of Residency p<0.001**	Northeast (A)	112	**80%**	B(<0.001) C(<0.001)
	Midwest (B)	125	55%	
	South (C)	65	51%	
	West (D)	105	**79%**	B(<0.001) C(<0.001)
**Current Religion p<0.001**	No religion identified (A)	77	**80%**	E(<0.001) F(<0.001)
	Atheist/Agnostic (B)	90	**87%**	E(<0.001) F(<0.001)
	Other (C)	39	74%	F(0.030)
	Jewish (D)	28	**86%**	E(0.002) F(<0.001)
	Catholic (E)	76	49%	
	Christian (all denominations) (F)	98	46%	
**Family’s Degree of Religiosity p<0.001**	Parents non-religious/Not at all (A)	57	**87%**	D(<0.001)
	Parents only attend during major holidays (B)	110	**78%**	D(<0.001)
	Parents are not practicing religion but do believe (C)	53	**73%**	D(<0.001)
	Yes, both parents attend services regularly (D)	186	53%	
**Current Religious Practice p<0.001**	Not at all (A)	202	**82%**	B(<0.001) C(<0.001)
	Only on major holidays (B)	140	57%	
	Actively (attend daily/weekly) (C)	65	42%	
**Political Party Affiliation p<0.001**	Democratic (A)	358	**73%**	B(0.002) C(<0.001)
	No Answer (B)	17	33%	
	Republican (C)	34	23%	
**Chose OBGYN because of "social aspects" p = 0.010**	No (A)	210	61%	
	Yes (B)	199	**74%**	A(0.010)
**Hospital Religious Affiliation p = 0.008**	Not religiously affiliated/none (A)	339	**70%**	B(0.006)
	Christian/Adventist/Baptist/Catholic (B)	61	49%	
	Jewish (C)	6	67%	
**Importance of Family Planning in Choosing a Residency p<0.001**	Chose because did NOT (A)	3	0%	
	Not at all important (B)	39	33%	
	Slightly or Moderately (C)	113	42%	
	Very or Extremely (D)	253	**84%**	A(0.003) B(<0.001) C(<0.001)
**Presence of a Ryan Program at your Residency? p<0.001**	No (A)	95	58%	
	Unsure (B)	30	40%	
	Yes (C)	263	**74%**	A(0.011) B(<0.001)
**Fraction of Faculty Provide Abortions p<0.001**	None (A)	41	34%	
	Only a few (B)	180	62%	
	About half (C)	103	**82%**	A(<0.001)
	Majority (D)	65	**80%**	A(0.018)
**First Trimester Medical Abortions (cases in past 6 mo) p<0.001**	No/none (A)	138	51%	
	1–5 (B)	128	73%	
	6–10 (C)	53	**81%**	A(0.021)
	11–20 (D)	39	79%	
	21+ (E)	29	79%	
**First Trimester Surgical Abortions (cases in past 6 mo) p<0.001**	No/none (A)	90	38%	
	1–5 (B)	108	67%	
	6–10 (C)	62	**79%**	A(0.032)
	11–20 (D)	70	**80%**	A(0.016)
	21+ (E)	58	**86%**	A(0.001)
**Surgical up to 18w Abortions (cases in past 6 mo) p<0.001**	No/none (A)	126	46%	
	1–5 (B)	154	**72%**	A(<0.001)
	6–10 (C)	55	**87%**	A(0.001)
	11–20 (D)	35	**91%**	A(0.002)
	21+ (E)	18	**78%**	A(0.011)
**Surgical up to 23w Abortions (cases in past 6 mo) p<0.001**	No/none (A)	187	51%	
	1–5 (B)	130	**80%**	A(<0.001)
	6–10 (C)	36	**92%**	A(<0.001)
	11–20 (D)	20	74%	
	21+ (E)	15	**93%**	A(0.029)
**Sought Out Additional Family Planning Training Outside? p<0.001**	No (A)	349	64%	
	Yes (B)	40	100%	A(<0.001)

Residents who intended to provide abortions post-training were compared to their peers.

In picking a program to match, those who intended to provide were more likely to choose a hospital that was not religiously affiliated (p = 0.008), considered it very or extremely important (p<0.001) that the program offers family planning, and were significantly more likely to join a residency with a Ryan program (p<0.001). Although there were no differences in number of contraceptive visits, tubal ligations performed, IUD and Nexplanon insertions between those intending and those not intending to provide, those in the former group reported having done more first trimester surgical abortions (p<0.004), abortions up to 18 weeks (p<0.001) and up to 23 weeks (p<0.001) than their peers. Residents intending to become providers also reported having sought out additional family planning training outside their home institution (p<0.001).

### Regression analysis

**Fig 1:**
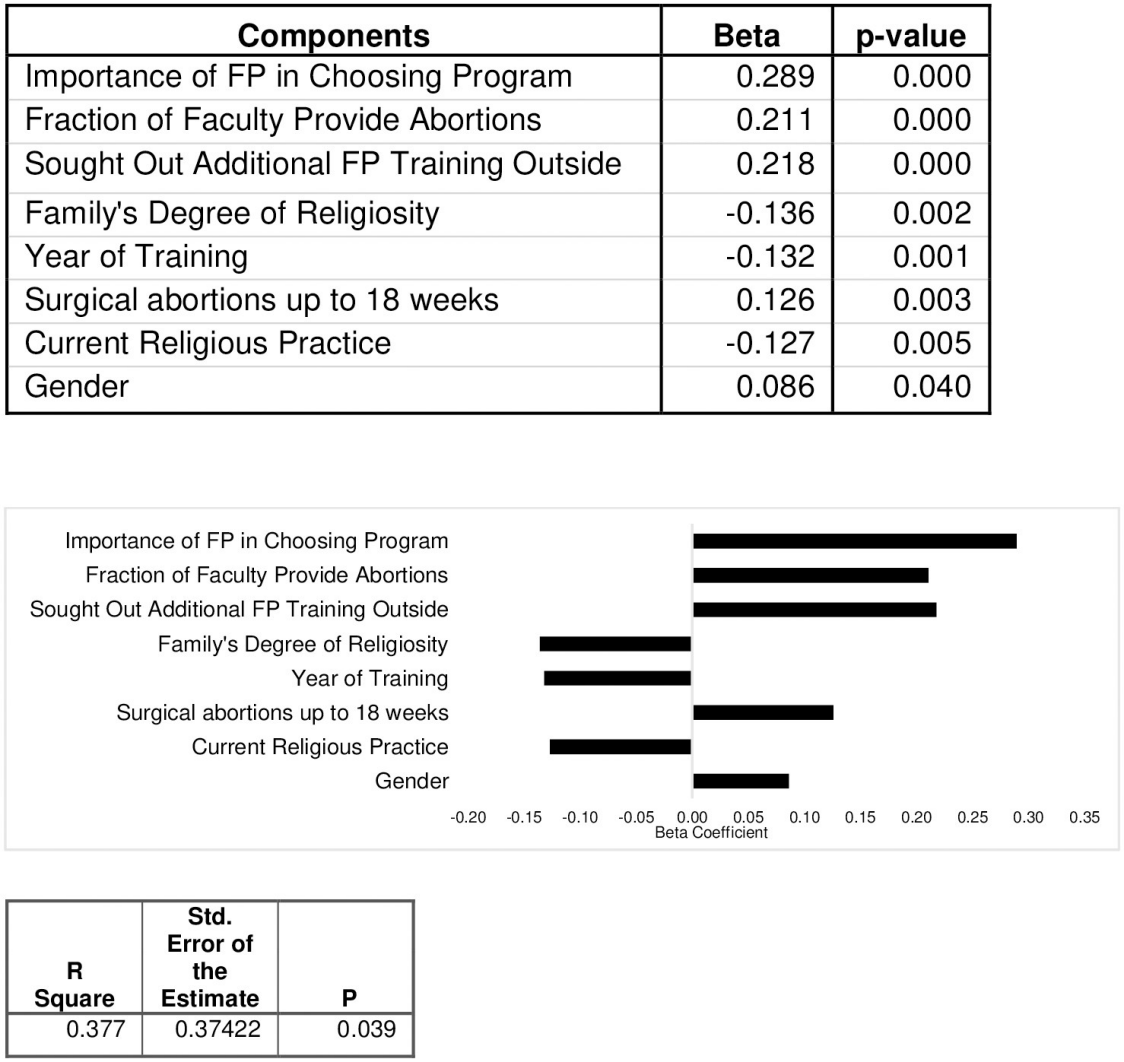
Stepwise regression. The model selected factors most correlated with intention to provide.

We performed a stepwise linear regression, Fig 1, and found that the highest predictors to intent to provide were the weight given by residents to choosing a program with strong family planning training opportunities (β = 0.289), the fraction of the faculty providing abortions (β = 0.211), the number of 2^nd^ trimester abortions performed (β = 0.126) and female gender (β = 0.086). Conversely, the family’s degree of religiosity (β = -0.136), the current religious practice (β = -0.127) and the year of training (β = -0.132) were inversely related to intent to provide. Our regression had an R^2^ = 0.377.

### Components of intent to provide

Having determined that intent to provide was significantly influenced by gender, religion, year of training and training environment, we examined each component individually and studied their effects and interrelation to one another.

#### Effect of gender

Sexual orientation was not independent of gender, as females identified as heterosexual more than males (p<0.001). Female residents tended to identify more often as Democrats (p<0.001) than their male counterparts, even when adjusted for geographical area of upbringing and religion. In selecting a residency to match, females were more likely to consider the importance of family planning as “extremely important” or “very important” (p = 0.024), S1 Table.

#### Effect of the current religious practice

Residents current religious practice was closely tied to their family’s degree of religiosity (p<0.001), however the younger generation tended to be less religious than their parents. Religion was tied to geography as more conservative religions, such as Catholics, were concentrated in the South and more liberal religions, such as Jewish and those not identifying as religious, in the Northeast and West respectively (p < .013). Residents who were not religious tended to perform higher volumes of abortions and considered it extremely or very important that a program offers family planning training, S2 Table.

#### Effect of year of training

The percentage of residents intending to provide for each class year and geographical area of training was analyzed. In both the Midwest and South, intent to provide declined significantly (p<0.001) from year one to year four of residency, S1 Fig.

#### Effect of faculty

Faculties having a high number of attendings performing abortions tended to be in the West and Northeast compared to other regions (p<0.001). Faculties having no attendings performing abortions tended to be at Christian affiliated hospitals, at institutions without Ryan Programs, and at programs significantly less likely to have an opt-out policy (p<0.001). In faculties which had over half of attendings offering abortions, case numbers were higher for medical (p<0.001) and surgical (p<0.001) abortions and dilation and evacuation was the method more often performed when compared to faculties with no abortion providers, who tended to favor induction or referral (p<0.001) as their dominant method of abortion. Residents who considered it very/extremely important to train at a program with family planning experience chose programs where a majority of faculty were abortion providers (p<0.01), S3 Table.

### Prediction model equation for intent to provide

**Fig 2:**
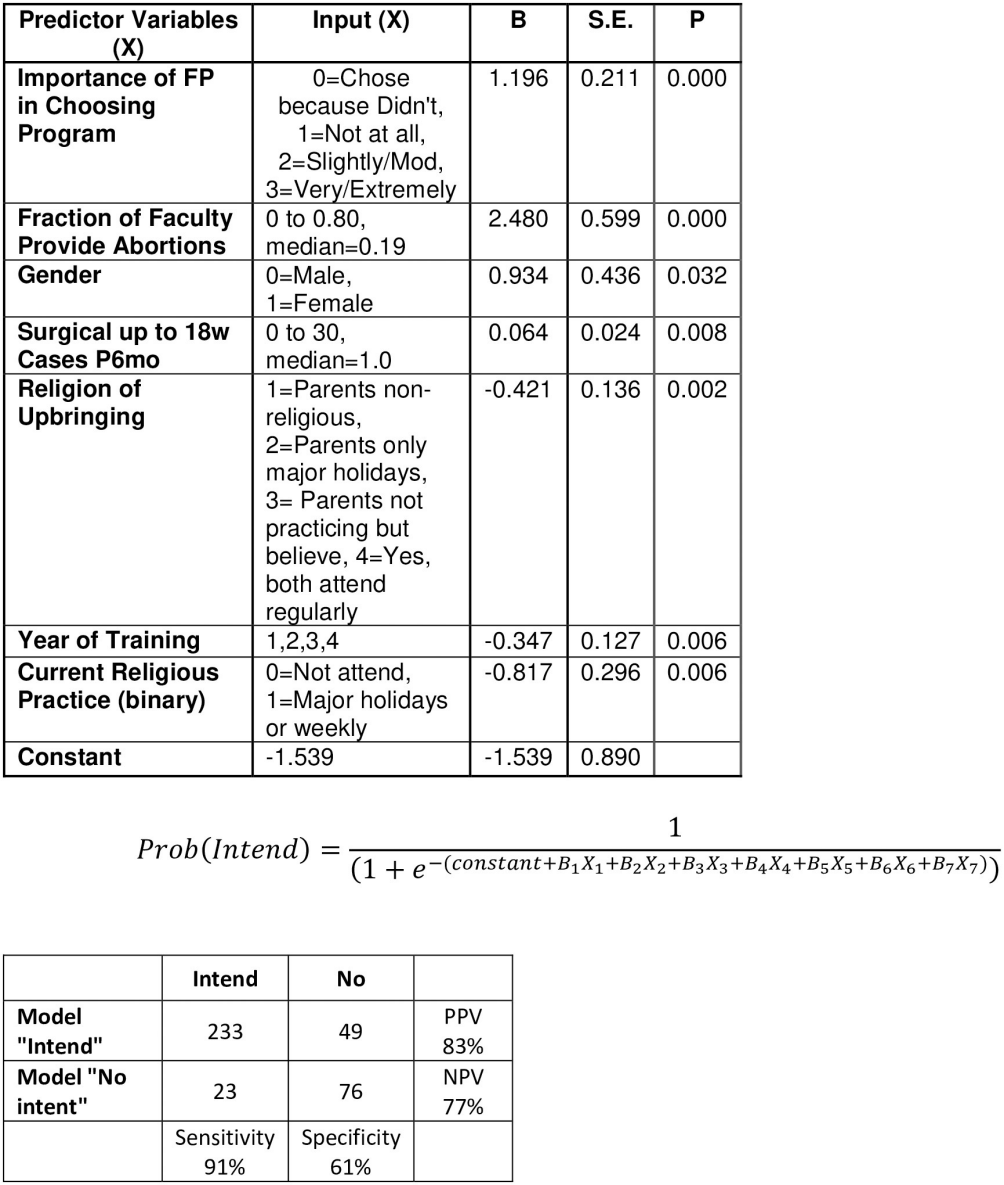
Prediction model for intention to provide.

Using our binary logistic regression analysis, we determined the unstandardized coefficients (“B”) for each component of the predictive model, Fig 2. The final model included seven predictor variables, each with a significance value of p<0.05 and had a Nagelkerke R^2^ = 0.377, a positive predictive value of 83%, a negative predictive value of 77%, a sensitivity of 91% and a specificity of 61%.

## Discussion

In this cohort, we found that 67% of residents intend to provide abortion after graduation, comparable but somewhat higher to other recent studies which were near 57% [12]. A review of IPA rates suggests that more residents are intending to offer abortions now than in the past, where rates ranged from 47%-39% in the early 2000’s [13, 14] to 30% in 1996 [15]. Compared with other countries, IPA was higher in the United States [16, 17]. This may be due to changes in the abortion climate, residency factors, personal attitudes and religiosity.

The strongest predictor of IPA was a medical student’s emphasis on the importance of having family planning training, a marker of pre-residency intention to provide. When applying to residencies, candidates who considered it “very” or “extremely” important that a program has family planning were significantly more likely to intend to provide, while those who stated that this was “not important” or “moderately important” were less likely to IPA (p<0.001), and many in this group denied any intention to provide at all. This finding is similar to those previously published [13]. Multiple other factors influenced residency selection by medical students [18], including diversity of clinical settings, surgical specialty, working with a female patient population and social aspects of care. In our analysis, those who considered the latter choice were also significantly more likely to provide abortions (p = 0.010) though these factors were much weaker predictors.

The volume of cases performed during training, particularly 2^nd^ trimester abortions, was also predictive of IPA. Residents who had performed a higher number of cases were more likely to intend to become providers, confirming other studies [13, 14, 19]. Residents who are trained and competent in abortion provision, through a sufficient number of procedures [20], are more likely to offer the procedure post-graduation. Other investigators have noted that exposure to abortion training is independently correlated with future provision [21]. In contrast to other studies [14], those with intent to provide were more likely to seek out training (p<0.001). This may be explained by the fact that those interested in providing abortion choose to take extra family planning electives to strengthen their dilation and evacuation skills [22] or audition at institutions offering complex family planning fellowships.

In addition to family planning case numbers, we found that the program’s abortion climate, especially the percentage of faculty members who perform abortions, was predictive of a resident’s future intentions (p<0.001). The faculty’s commitment to abortion training [19] and the integration of abortion as part of routine practice [22] not only translated to higher number of abortion cases (p<0.001) but also to teaching advanced procedures such as dilation and evacuation (p<0.001). Programs with few providers relied only on induction or referrals (p<0.001). Our results showed that residents at institutions with Ryan Programs tended to have higher IPA (p<0.001), perhaps due to program and faculty support for abortion training and higher case volumes. Furthermore, resident applicants with a strong pre-residency IPA may be more attracted to match at institutions with Ryan programs [23]. Those intending to perform abortions were more likely to be in opt-out programs (p<0.001). We found few instances of self-perceived stigmatization of residents who refused to participate in training.

Our model also suggested that IPA rates decreased from internship through senior year in programs in the South and Midwest (p<0.001). Possibly, residents who came into the specialty intending to provide felt that they were not adequately trained, encountered political, social or legal backlash, or chose to focus their careers on subspecialties not typically providing abortions.

Religion influenced IPA, confirming other studies [23]. Religiosity was measured by three metrics: the religion the resident identifies with, the parent’s degree of religiosity (as measured by regular attendance) and the resident’s personal degree of participation in religious practice. These metrics correlated with one another and the degree of religiosity entered the regression as an important negative correlate of abortion provision (p<0.001). This finding is mirrored in other studies that showed that Catholics, Evangelicals and physicians with high religious beliefs were less likely to provide abortions or provide a referral [24] as residents [13] and as attendings [21].

Geography of residency training impacted intent to provide (p<0.001) as those in the West and those in the Northeast had higher IPA. These two regions tended to have a higher prevalence of more liberal religions [9] such as no-religion (in the West) or Jewish (in the Northeast) while individuals from more conservative religions such as Catholics and Christians were in the Midwest and South respectively, validating other papers [25]. Our study also confirmed that personal characteristics, such as female gender [24] were correlated with IPA.

Based on our regression, we established a model with excellent accuracy for predicting intenders, but only moderate accuracy for predicting non-intenders. The predicted probability cutoff value (currently 0.5) can be changed to lower the false positives while increasing the false negatives. Such a model may be used by program directors and Ryan program attendings in the future.

One of the limiting factors in the study was the sample size, representing about a 10% response rate and the potential selection bias incurred by having program directors forward the survey invitation to residents. A selection bias from those who chose to participate may also affect results. While 68% of respondents were training at Ryan sites, about half (48%) of all U.S. residency spots were in Ryan Programs, indicating a skew. Another weaknesses in the study is that the survey did not adequately address issues such as race [12] which may play a key role in the provision of abortion. The survey was also not designed as a longitudinal study, making it difficult to draw conclusions regarding the evolution of a resident’s intent to provide over the time course of the residency program. While the survey was answered by a geographically and demographically diverse group of residents, the results may have been impacted by the skew in gender (89%). Some residents chose not to answer every question which may represent recall or personal biases.

Additionally, the survey only assesses intentions after residency and not actual practice which can be as low as 3–14% [24] as multiple barriers may prevent the integration of abortion into practice [26]. With the political climate surrounding abortion in the United States, new legal and insurance reimbursement barriers may emerge in the near future. While the scope of this paper is limited to examining factors that influence a resident’s IPA, the authors recognize that intent to provide and actual provision of abortion in practice are different. Additionally, the questionnaire did not address reasons behind non-intention, such as pursuing subspecialties not typically providing family planning.

## Conclusion

In conclusion, residents who have a high pre-residency intention on providing abortions, who consider it important to match at a program with family planning training, who sought out additional training, who have performed a substantial number of procedures, and who are female are more likely to intend to become abortion providers. Conversely, those from a religious background are least likely to intend to provide abortions. One finding in our study showed that, for residents training in the South or Midwest, intent declined over the years of training, suggesting that culture and environment can modulate intention rates. Residency programs may play a role in increasing IPA, and potentially improving abortion access, by expanding abortion training volumes, establishing relationships with freestanding clinics, hosting a Ryan Program and hiring faculty who perform family planning procedures. With the reversal *Roe v Wade* and *Dobbs v*. *Jackson*, those intending to provide abortions will have to overcome multiple barriers [12, 13, 21]. Further research must focus on studying the impact of this ruling on training sites, clinical training opportunities and intention to provide.

## Supporting information

S1 File Family planning survey entered into Qualtrix. A link was sent to program directors to pass onto their residents.
(DOC)

S1 Fig Effect of region and year of training. (TIF)

S1 Table Effect of gender. (DOCX)

S2 Table Effect of current religious practice. (DOCX)

S3 Table Effect of faculty. (DOCX)
