# Supplementary material for: Predictors of intention to provide abortions after OB/GYN residency training
Source: PLoS One. 2023 Jun 29;18(6):e0286703. doi: 10.1371/journal.pone.0286703 (PMC10309643; doi:10.1371/journal.pone.0286703)
Supplement: S1 File — A link was sent to program directors to pass onto their residents. (DOC) [file pone.0286703.s001.doc]

**S1 Fig. Family Planning Survey entered into Qualtrix.** A link was sent to program directors to pass onto their residents.

**Research Information Sheet**

**Survey of Ob-Gyn Residents’ Family Planning Experiences**

**Principal Investigator (PI):** Maurice-Andre Recanati, MD and Aliye Runyan, MD

Department of Obstetrics and Gynecology; Wayne State University, Detroit, MI

**Purpose**: You are being asked to be in a research study of ob-gyn residents’ experiences in family planning training because you are currently in an ob-gyn residency training program. The aim of the study is to gather information to potentially help to improve the quality and standardization of family planning within ob-gyn residency programs. This study is being conducted at Wayne State University.

**Study Procedures**: If you take part in the study, you will be asked to fill out the following survey linked in this email. The questions are designed to get a sense of your background and the breadth and depth of family planning training within your residency program. The survey questions are voluntary.

**Bene**fi**ts**: As a participant in this research study, there may be no direct benefit for you; however, information from this study may benefit other people now or in the future.

**Risks**: There are no known risks at this time to participation in this study.

**Costs**: There will be no costs to you for participation in this research study.

**Compensation**: **Respondents will be randomly selected to receive a Starbucks gift card**

**Con**fi**dentiality**: All information collected about you during the course of this study will be kept without any identifiers.

**Voluntary Participation /Withdrawal**: Taking part in this study is voluntary. You are free to not answer any questions or withdraw at any time.

**Questions**: If you have any questions about this study now or in the future, you may contact [Aliye Runyan] at the following phone number []. If you have questions or concerns about your rights as a research participant, the Chair of the Institutional Review Board can be contacted at (313) 577-1628. If you are unable to contact the research staff, or if you want to talk to someone other than the research staff, you may also call the Wayne State Research Subject Advocate at (313) 577-1628 to discuss problems, obtain information, or offer input. Participation: By completing the survey you are agreeing to participate in this study. The data that you provide may be collected and used by Qualtrics as per its privacy

**Demographics**

Age:

1. 25-30
2. 30-35
3. 36-45
4. 46 and over
5. Decline

Gender

1. Male
2. Female
3. Transgender/Intersex/Non-binary
4. Decline

Sexual orientation

1. Heterosexual
2. Homosexual
3. Bisexual
4. Other
5. Decline

Marital Status

1. Single
2. Married
3. Engaged
4. Dating
5. Long term partner
6. Decline

State where you grew up:

Size of city or town:

1. Major metropolitan area of 1 million people or greater

2. Large city of half million to 1 million people

3. City of 100,000 to half million people

4. Small urban area of 50,000 to 100,000 people

5. Rural (less than 50,000)

6. Decline

State of residency training:

Year of Training

1. PGY1
2. PGY2
3. PGY3
4. PGY4

**Religious background**

Religion you identify with:

1. Jewish
2. Catholic
3. Baptist
4. Evangelical
5. Protestant
6. Nondenominational Christian
7. Muslim
8. Buddhist
9. Hindu
10. Sikh
11. Atheist/Agnostic
12. Other
13. No religion identified

Did religion play a large role in your upbringing?

1. Yes, both parents attend services regularly
2. Parents only attend during major holidays
3. Parents are not practicing religion but do believe
4. Parents nonreligious/ Not at all

Do you currently practice:

1. Actively (attend daily/weekly)
2. Only on major holidays
3. Not at all

**Political views**

Which candidate did you support in the last election cycle?

1. Clinton
2. Sanders
3. Trump
4. Rubio/Cruz

**Residency program**

What factors made you decide to choose a career in obgyn (choose top 3 that apply)?

1. Diversity of settings (OR, clinic, L&D)
2. Female patient population
3. Primary care specialty
4. Surgical specialty
5. social aspects of care

Hospital Affiliation with Religious group?

1. Christian/Adventist/Baptist/Catholic
2. Jewish
3. Not religiously affiliated/None

When matching residency how important was it that the program provided family planning training?

1. Extremely important
2. Very important
3. Moderately important
4. Slightly important
5. Not at all important
6. Chose program because it did not offer family planning

Is your program a Ryan Residency program?

1. Yes
2. No
3. Unknown

Does your program provide opt-in or opt-out family planning training?

1. Opt-In
2. Opt-Out

Is there a stigma for those who opt out of providing family planning?

1. Yes
2. No, we have residents who choose to opt out without issues

Does your program provide training on contraceptive methods? (if so, how many contraceptive visits have you seen during the last 6 months)

1. no/none
2. 1-5
3. 6-10
4. 11-20
5. >20

Does your program provide training on tubal ligation? (If yes, how many cases have you done by any method over the last 6 months?)

1. no/none
2. 1-5
3. 6-10
4. 11-20
5. >20

Does your program provide training on IUD insertion? (If yes, how many cases have you done in the last 6 months?)

1. no/none
2. 1-5
3. 6-10
4. 11-20
5. >20

Does your program provide training on Nexplanon insertion? (If yes, how many cases have you done in the last 6 months?)

1. no/none
2. 1-5
3. 6-10
4. 11-20
5. >20

Does your program provide experience with medical abortion? (If yes, how many cases have you done in the last 6 months?)

1. no/none
2. 1-5
3. 6-10
4. 11-20
5. >20

Does your program provide experience with 1^st^ trimester surgical abortion and if so how many cases have you done in the last 6 months?

1. no/none
2. 1-5
3. 6-10
4. 11-20
5. >20

Does your program provide experience with surgical abortion up to 18 weeks and if so how many cases have you done in the last 6 months?

1. no/none
2. 1-5
3. 6-10
4. 11-20
5. >20

Does your program provide experience with surgical abortion up to 23 weeks and if so how many cases have you done in the last 6 months?

1. no/none
2. 1-5
3. 6-10
4. 11-20
5. >20

Does your program provide experience with surgical abortion up term if indicated and if so how many cases have you done in the last 6 months?

1. no/none
2. 1-5
3. 6-10
4. 11-20
5. >20

Does your program provide training on options counseling

1. Yes, in formal didactics
2. Only as needed in the clinic
3. Not at all

Delineate where the abortion experiences come from?

1. Hospital
2. abortion clinic during a separate rotation
3. regular or continuity clinic

Have you sought out additional family planning training outside of your program?

1. Yes
2. No

What percentage of faculty members provide abortions?

1. Majority
2. About half
3. only a few
4. none

What method of termination is routinely used in the 2^nd^ trimester at your institution (lethal abnormalities or elective)?

1. D&E
2. Induction
3. Referral to another facility

Are you planning to offer family planning services after graduation?

1. Yes, only medical abortion
2. Yes, first trimestr abortion
3. Yes, second trimester abortion
4. Yes, second trimester abortion after additional training
5. No

Optional questions:

What method of birth control do you use?

1. Hormonal (OCPs, NuvaRing, Patch, DepoProvera)
2. Nexplanon
3. Progestin IUD
4. Paragard IUD
5. Condoms
6. None
7. Other
8. Decline

Have you or someone close to you ever had an abortion?

1. yes
2. no

Please include your email address if you would like to be entered into the random drawing for a Starbucks card.
