# Supplementary figures and images for: Predictors of intention to provide abortions after OB/GYN residency training
Source: PLoS One. 2023 Jun 29;18(6):e0286703. doi: 10.1371/journal.pone.0286703 (PMC10309643; doi:10.1371/journal.pone.0286703)

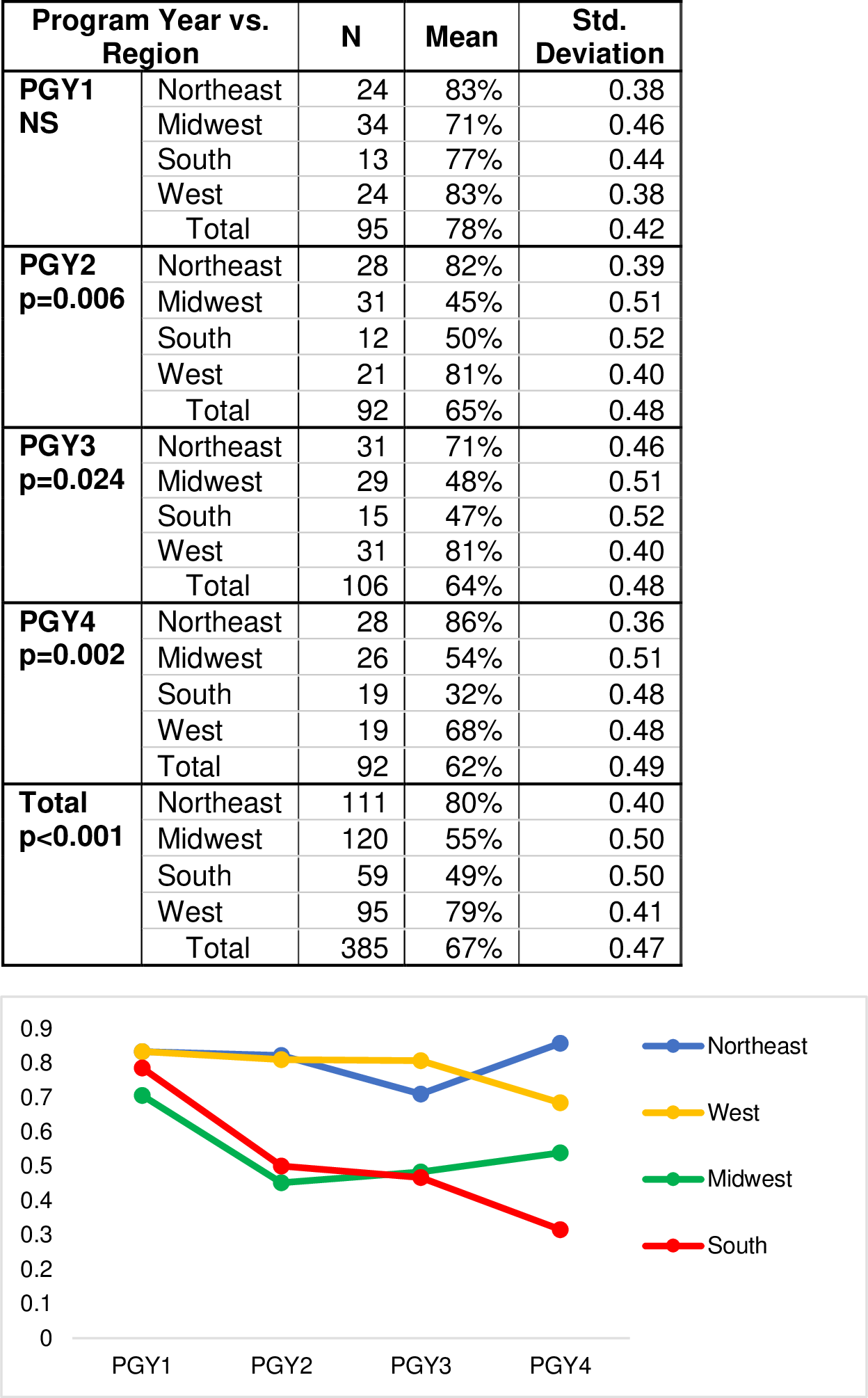

Supplement: S1 Fig — (TIF) [file pone.0286703.s002.tif]
