# Supplementary material for: Predictors of intention to provide abortions after OB/GYN residency training
Source: PLoS One. 2023 Jun 29;18(6):e0286703. doi: 10.1371/journal.pone.0286703 (PMC10309643; doi:10.1371/journal.pone.0286703)
Supplement: S1 Table — (DOCX) [file pone.0286703.s003.docx]

|  | | **Gender** | | | | | |
| --- | --- | --- | --- | --- | --- | --- | --- |
|  |  | **Male** | | | **Female** | | |
|  |  | Count (%) | row % | p-value | Count (%) | row % | p-value |
| **Sexual Orientation p<0.001** | Heterosexual (A) | 29 (67) | 8% |  | 340 (94) | 92% | C(<0.001) |
|  | Bisexual (B) | 4 (9) | 21% |  | 15 (4) | 79% | C(<0.001) |
|  | Homosexual (C) | **10 (23)** | 67% | A(<0.001) B(<0.001) | **5 (2)** | 33% |  |
| **Religion of Upbringing p<0.032** | Parents non religious/ Not at all | 3 (7) | 5% |  | 54 (15) | 95% |  |
|  | Parents only attend during major holidays | 6 (14) | 5% |  | 2 (29) | 95% |  |
|  | Parents are not practicing religion but do believe | 8 (18) | 15% |  | 45 (12) | 85% |  |
|  | Yes, both parents attend services regularly | 27 (61) | 15% |  | 158 (44) | 85% |  |
| **Political Party Affiliation p<0.001** | Democratic (A) | 31 (69) | **9%** |  | 326 (90) | **91%** | B(0.001) C(0.022) |
|  | No Answer (B) | 6 (13) | 38% | A(0.001) | 10 (3) | 63% |  |
|  | Republican (C) | 8 (18) | 24% | A(0.022) | 26 (7) | 76% |  |
| **Importance of FP in Choosing Program p=0.024** | Chose because it Didn't (A) | 0 | 0% |  | 3 (1) | 100% |  |
|  | Not at all important (B) | 11 (24) | **28%** | C(0.023) D(0.001) | 28 (8) | 72% |  |
|  | Slightly or Moderately (C) | 13 (29) | 12% |  | 100 (28) | 88% | B(0.023) |
|  | Very or Extremely (D) | 21 (47) | 8% |  | 231 (64) | **91%** | B(0.001) |

**S1 Table. Effect of gender.**
