# Supplementary material for: Predictors of intention to provide abortions after OB/GYN residency training
Source: PLoS One. 2023 Jun 29;18(6):e0286703. doi: 10.1371/journal.pone.0286703 (PMC10309643; doi:10.1371/journal.pone.0286703)
Supplement: S2 Table — (DOCX) [file pone.0286703.s004.docx]

|  | | **Current Religious Practice** | | | |  |
| --- | --- | --- | --- | --- | --- | --- |
|  |  | **None (A)** | | **Attend major holidays or weekly (B)** | | **p-value** |
|  |  | Count (%) | Row % | Count (%) | Row % |  |
| **Importance of Family Planning in Choosing Program p<0.001** | Chose because it Didn't | 0 | 0% | 3 (1) | 100% |  |
|  | Not at all important | **9 (4)** | 23% | 30 (15) | 77% | BA(<0.001) |
|  | Slightly or Moderately | 35 (17) | 31% | **77 (38)** | 69% | BA(<0.001) |
|  | Very or Extremely | **158 (78)** | 62% | 95 (46) | 38% | AB(<0.001) |
| **Family’s degree of Religiosity p<0.001** | Parents non-religious/ Not at all | 48 (24) | 84% | **9 (4)** | 16% | AB(<0.001) |
|  | Parents only attend during major holidays | 56 (28) | 51% | 54 (26) | 49% |  |
|  | Parents are not practicing religion but do believe | 42 (21) | 81% | **10 (5)** | 19% | AB(<0.001) |
|  | Yes, both parents attend services regularly | 55 (27) | 30% | **131 (64)** | 70% | BA(<0.001) |
| **Training Tubal (Cases in past 6 months) p=0.035** | mean | 3.1 | | **3.3** | | BA(0.035) |
| **First Trimester Surgical (Cases in past 6 months) p=0.026** | mean | **2.9** | | 2.6 | | AB(0.026) |

**S2 Table. Effect of current religious practice.**
