# Supplementary material for: Predictors of intention to provide abortions after OB/GYN residency training
Source: PLoS One. 2023 Jun 29;18(6):e0286703. doi: 10.1371/journal.pone.0286703 (PMC10309643; doi:10.1371/journal.pone.0286703)
Supplement: S3 Table — (DOCX) [file pone.0286703.s005.docx]

|  |  | **Fraction of Faculty Who Perform Abortions** | | | | | | **p-value** |
| --- | --- | --- | --- | --- | --- | --- | --- | --- |
|  |  | **None (A)** | | **Only a Few (B)** | | **Half or a Majority (C)** | |  |
|  |  | Count (%) | Row % | Count (%) | Row % | Count (%) | Row % |  |
| **Current Religious Practice p=0.048** | None | **13 (33)** | 7% | 94 (53) | 48% | **90 (54)** | 46% | CA(0.005) |
|  | Attend major holidays or weekly | **27 (66)** | 14% | 85 (47) | 45% | **78 (46)** | 41% | AC(0.005) |
| **Hospital Religious Affiliation p<0.001** | Not religiously affiliated/none | **21 (53)** | 6% | 151 (85) | 46% | 153 (94) | 47% | BA(<0.001) CA(<0.001) |
|  | Christian/ Adventist/ Baptist/ Catholic | **19 (48)** | 35% | 26 (15) | 47% | 10 (6) | 18% | AB(<0.001) AC(<0.001) BC(0.028) |
|  | Jewish | 0 | 0% | 2 | 33% | 4 | 67% |  |
| **Importance of Family Planning in Choosing Program p=0.010** | Chose because it Didn't | 1 (3) | 33% | 1 (1) | 33% | 1 (1) | 33% |  |
|  | Not at all important | 6 (15) | 17% | 2 (8) | 42% | 15 (9) | 42% |  |
|  | Slightly or Moderately | 18 (45) | **17%** | 52 (29) | 50% | 34 (20) | 33% | AC(0.003) |
|  | Very or Extremely | 15 (38) | **6%** | 112 (62) | 46% | 118 (20) | 48% | BA(0.013) CA(<0.001) |
| **Is Residency a Ryan Program? P<0.001** | No | **26 (63)** | 27% | 40 (22) | 42% | 29 (17) | 31% | AB(<0.001) AC(<0.001) |
|  | Unsure | 11 (27) | 37% | 13 (7) | 43% | 6 (4) | 20% | AB(0.001) AC(<0.001) |
|  | Yes | **4 (10)** | 2% | 127 (71) | 48% | 132 (79) | 50% | BA(<0.001) CA(<0.001) |
| **Opt-In or Opt-Out Program p<0.001** | Opt-In | **16 (39)** | 39% | 14 (8) | 34% | 11 (7) | 27% | AB(<0.001) AC(<0.001) |
|  | Opt-Out | **25 (61)** | 7% | 166 (92) | 48% | 157 (93) | 45% | BA(<0.001) CA(<0.001) |
| **Training 1st Tri Medical (Cases in past 6 months) p<0.001** | mean | 1.8 | | 5.9 | | **7.0** | | BA(0.014) CA(0.001) |
| **Training 1st Trimester Surgical Abortions (Cases in past 6 months) p<0.001** | mean | 2.2 | | 9.3 | | **11.5** | | BA(<0.001) CA(<0.001) |
| **Surgical abortions to <18** **weeks (Cases in past 6 months) p<0.001** | mean | 0.8 | | 4.9 | | **6.4** | | BA(0.014) CA(0.001) |
| **Surgical cases <23w (Cases in past 6 months) p<0.001** | mean | 0.5 | | 3.4 | | **4.9** | | BA(<0.001) CA(<0.001) |
| **2nd Trimester Method - D&E p<0.001** | % | 20% | | 83% | | **96%** | | BA(<0.001) CA(<0.001) CB(<0.001) |
| **2nd Trimester Method - Induction p<0.001** | % | **29%** | | 61% | | 65% | | BA(<0.001) CA(<0.001) |
| **2nd Trimester Method - Referral p<0.001** | % | **73%** | | 18% | | 6% | | AB(<0.001) AC(<0.001) BC(0.002) |

**S3 Table. Effect of faculty.**
